# SARS-CoV-2 and chronic myeloid leukemia: a systematic review

**DOI:** 10.3389/fmed.2023.1280271

**Published:** 2024-01-24

**Authors:** Elrazi A. Ali, Anas Al-Sadi, Qusai Al-maharmeh, Eihab A. Subahi, Amulya Bellamkonda, Madhumati Kalavar, Kalpana Panigrahi, Awni Alshurafa, Mohamed A. Yassin

**Affiliations:** ^1^Internal Medicine Department, Interfaith Medical Center/One Brooklyn Health, Brooklyn, NY, United States; ^2^Internal Medicine Department, Hamad Medical Corporation, Doha, Qatar; ^3^Internal Medicine Department, Saint Michael's Medical Center, Newark, CA, United States; ^4^Department of Oncology-Hematology, National Center for Cancer Care and Research – Hamad Medical Corporation, Doha, Qatar

**Keywords:** SARS-CoV-2 variants, COVID-19, SARS-CoV-2, chronic myelocytic leukemia (CML), chronic myeloid leukemia

## Abstract

**Introduction:**

Severe acute respiratory syndrome coronavirus 2 (SARS-CoV-2) is the virus causing the coronavirus disease of 2019. The disease has caused millions of deaths since the first pandemic at the end of 2019. Immunocompromised individuals are more likely to develop severe infections. Numerous mutations had developed in SARS-CoV-2, resulting in strains (Alfa Beta Delta Omicron) with varying degrees of virulence disease severity. In CML (chronic myeloid leukemia) patients, there is a lot of controversy regarding the effect of the treatment on the patient outcome. Some reports suggested potential better outcomes among patients with CML, likely due to the use of TKI; other reports showed no significant effects. Additionally, it is unknown how much protection immunization provides for cancer patients.

**Method:**

In accordance with the Preferred Reporting Items for Systematic Reviews and Meta-Analyses (PRISMA) standards, we conducted a systematic review. Retrospective, prospective studies, reviews, case series, and case reports of chronic myeloid leukemia patients aged above 18 years who had SARS-CoV-2 infection were included. English literature was screened using PubMed, SCOPUS, and Google Scholar. Search terms include chronic myeloid leukemia, chronic myelogenous leukemia, and SARS-CoV-2 and Coronavirus disease 2019 (COVID-19). We searched the reference lists of the included studies for any new articles. The search included all articles published up to April 20, 2023. The review is registered in PROSPERO (registration number CRD42022326674).

**Results:**

We reviewed 33 articles of available published literature up to April 2023 and collected data from a total of 682 CML patients with COVID-19. Most patients were in the chronic phase, seven were in the accelerated phase, and eight were in the blast phase. Disease severity was classified according to WHO criteria. Mortality was seen in 45 patients, and there were no reports of thrombotic events. Two hundred seventy-seven patients were in the era before vaccination; among them, eight were in the intensive care unit (ICU), and mortality was 30 (11%). There were 405 patients after the era of vaccination; among them, death was reported in 15 (4%) patients and ICU in 13 patients.

**Limitations and conclusion:**

The major limitation of this review is the lack of details about the use or hold of TKIs during SARS-CoV-2 infection. Additionally, after the appearance of the different variants of the SARS-CoV-2 virus, few studies mentioned the variant of the virus, which makes it difficult to compare the outcome of the other variants of the SARS-CoV-2 virus in patients with CML. Despite the limitations of the study, CML patients with COVID-19 have no significant increase in mortality compared to other hematological malignancy. Hematological cancers are associated with an increased risk of thrombosis, which is expected to increase in patients with COVID-19. However, patient with CML has not been reported to have a significant increase in thrombosis risk. The available data indicates that COVID-19’s effect on patients with chronic myeloid leukemia (CML) still needs to be better understood due to the limited data.

**Systematic review registration:**

https://www.crd.york.ac.uk/PROSPERO/display_record.php? RecordID:326674.

## Introduction

Chronic myeloid leukemia (CML) is a clonal myeloproliferative neoplasm with an overproduction of mature granulocytes. Nearly half of patients are asymptomatic and detected during routine screening or during routine blood work. Usually, patients present with abdominal distension, early satiety, and fatigue. Others may have atypical presentations and complications like priapism, eye symptoms or abdominal pain, and appendicitis (1–3). After the introduction of tyrosine kinase inhibitors, the treatment goals of CML have changed dramatically, and Patients with CML are expected to have a normal life expectancy, and treatment aims for a better quality of life (4). Worldwide, SARS-CoV-2 has caused millions of deaths. Mortality is higher for patients with multiple medical conditions, such as diabetes mellitus and hypertension (5, 6). Besides the respiratory manifestation, COVID-19 can present with liver renal cutaneous manifestations (7–9). Recently, the overall mortality of COVID-19 has improved with time due to improvements in preventive measures, the presence of vaccination, and effective antiviral medications. What the exact risk is for CML patients infected with SARS-CoV-2 is unclear, though. Additionally, the real mortality impact of immunization, ideal treatment options, and interactions with CML and COVID-19 therapy need to be explored. Many studies suggested a potentially higher risk of mortality among CML patients from COVID-19, while other studies reported lower mortality with a presumed protective effect from TKI. Additionally, the impact of SARS-CoV-2 on CML patients and the outcome with the different strains is poorly understood.

## Methods

Following the PRISMA (preferred reporting items for systematic reviews and meta-analyses) guidelines, PubMed, Scopus, and Google Scholar databases were searched for published articles through April 20, 2023, for qualified studies. The search included Retrospective, prospective studies, randomized control trial, reviews, case series, and case reports. The inclusion criteria were English literature with CML patients above 18 years who had SARS-CoV-2 infection. Articles in a language other than English articles about patients less than 18 years and articles with non-sufficient information were excluded, and patients with bone marrow transplants were excluded. Search terms were (chronic myeloid leukemia) OR (chronic myelogenous leukemia) AND (SARS-CoV-2) OR (COVID-19). Two independent reviewers (EA and A.E.) evaluated the studies for inclusion in the review by looking through the titles and abstracts of the search records they had retrieved to see which ones were eligible. If there was ever a disagreement between the independent reviewers over a research’s eligibility, it was usually resolved via careful consideration and evaluation of the study in question, a third reviewer, was consulted in case consensus could not be established. Excluded studies were those that did not fit the predetermined qualifying requirements. We searched the reference lists of the included studies for any new articles. The search included all articles published up to April 20, 2023. The review is registered in PROSPERO (registration number CRD42022326674).

## Results

**Figure 1:**
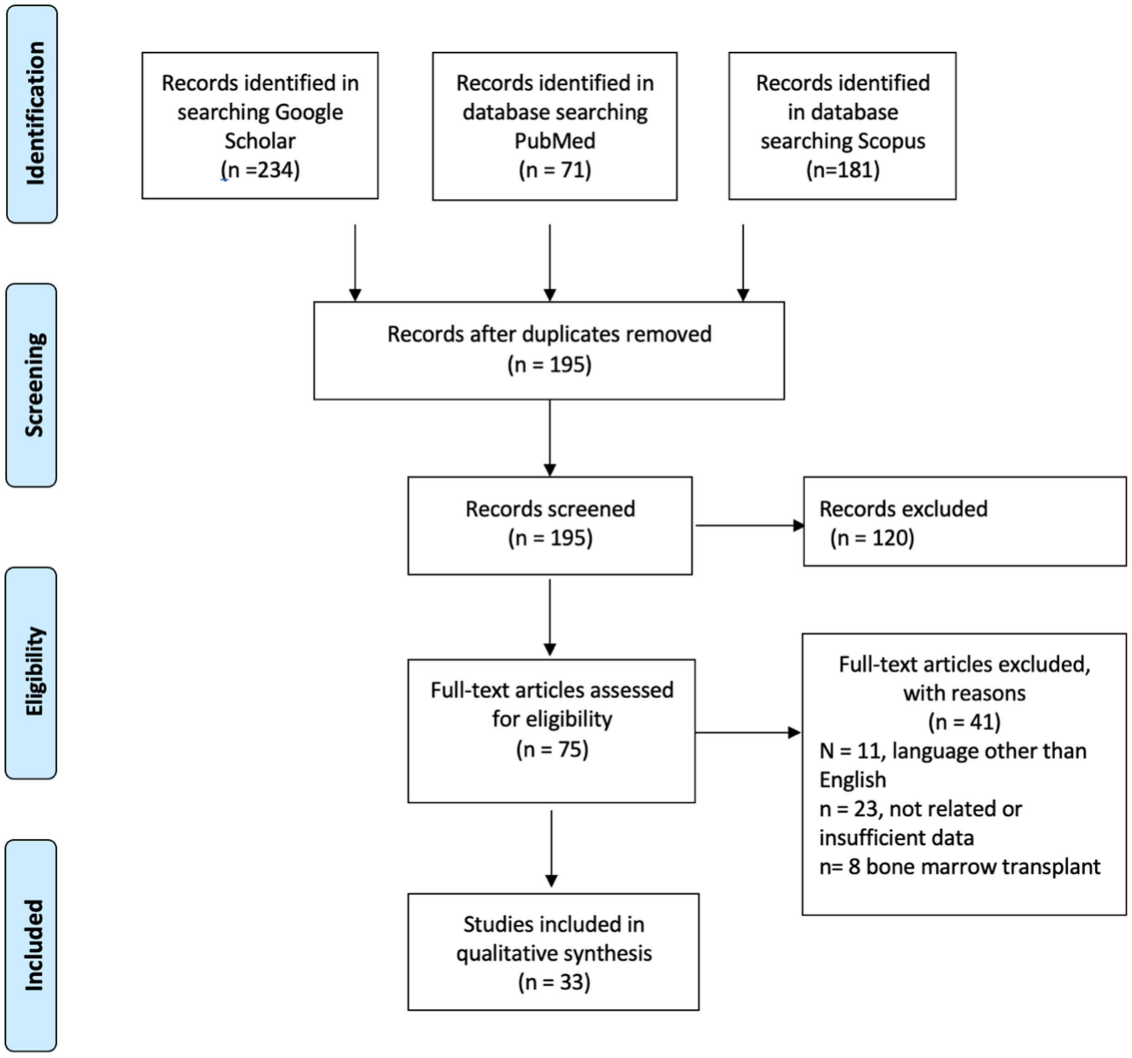
The PRISMA flow diagram detailing articles screening of chronic myleid leukemia patient with SARS-CoV-2.

We reviewed 33 articles of available published literature (Figure 1) up to April 2023 and collected data from a total of 682 CML patients with COVID-19 (35–70). Most of the reports were from the United States, Europe (United Kingdom, Italy, Germany), and China. Twenty-four studies were reported before and 12 after the introduction of vaccination. Most patients were in the chronic phase, seven patients were in the accelerated phase, and eight patients were in the blast phase. Disease severity was classified as per WHO criteria (10), severe disease if a patient requires oxygen or, has saturation below 94% or requires ventilatory support. SARS-CoV-2 infection was reported in both patients with a recent diagnosis of CML and in patients diagnosed with CML for months to years (up to 14 years). Twenty-seven patients were managed at home, and 21 patients were in ICU (intensive care unit) during the hospital course. Forty-one patients required oxygen therapy; among them, seven patients required mechanical intubation, and six patients required non-invasive ventilation. Mortality was seen in 45 patients, and there were no reports of thrombotic events. In the pre-vaccination era (Supplementary Tables 1, 2), five patients were intubated, four required NIV, and nine required oxygen. In the post-vaccination era (Supplementary Tables 1, 2), 32 (8.1%) patients out of 407 required oxygen; among them, two were intubated, and 3 required NIV. Two hundred seventy-seven patients were in the era before vaccination; among them, eight were in ICU, and mortality was 30 (11%). There were 405 patients after the era of vaccination; among them, death was reported in 15 (4%) patients and ICU in 13 (3.19%) patients. This is expressed as a Z score of 3.5614 with a *p* value of 0.00038. The result is significant at *p* < 0.05. For patients admitted to ICU, the mortality was 4/8 in the pre-vaccination era and 9/13 in the post-vaccination era. For the vast majority of the patients, there was no clear documentation if treatment of CML was continued or held during COVID-19. The first COVID-19 vaccine was the Pfizer-BioNTech COVID-19 Vaccine, which was first available on December 11, 2020, followed by other vaccines like Moderna. Also, there was no documentation about prior vaccination for cases reported after introducing COVID-19 vaccines. Many patients had comorbidities, including diabetes mellitus, hypertension, chronic kidney disease, coronary artery disease, hypothyroidism, obesity, dyslipidemia, prostate cancer, and gastroesophageal reflux disease. There was no report of thrombotic events; however, some patients developed AKI, autoimmune hemolytic anemia, rhabdomyolysis, DIC and possible HLH, cranial nerve palsy, and DKA during COVID-19.

## Discussion

The primary factor in mortality from COVID-19 is often ARDS; other factors include cardiac arrhythmia, cardiac arrest, and pulmonary embolism. The average mortality for the general population from COVID-19 varies; during the first wave, there were no vaccines, and the mortality was around 4.3% (11). However, after the emergence of vaccination, worldwide mortality went down. The availability of resources plays a major role; in limited-resource countries, the mortality was reported to be extremely high, up to 48% (12). Patients with hematological malignancies have a high rate of mortality with SARS-CoV-2 infection. In a study with over 300 hematology patients (70% myeloma, acute leukemia, and active lymphoma), the mortality was 26%, which is markedly higher than our findings in CML patients (13). The same study showed a mortality of 83% for patients with critical illness. Our review showed that for ICU-admitted CML patients, the mortality exceeds 50%: 4/8 (50%) in the pre-vaccination era and 9/13 (69%) in the post-vaccination era. This data showed a three-times drop in overall mortality in CML patients in the post-vaccination era, 11 to 4%, but high ICU mortality. This would suggest that COVID-19 immunization and treatment are effective in reducing illness severity but have little impact on lowering fatality rates for critical patients with CML. On the other hand, mortality in the general population varied significantly during the different waves of the pandemic, but the ICU mortality did not change substantially; most reports show mortality around 23 to 28% (14).

Fortunately, the majority of SARS-CoV-2 mutations have no effect on viral function. Only a few mutations resulted in new variants with significant effects on transmission and clinical implications on populations. It resulted in different strains, some of which have increased spread but low severity of illness like Omicron (Table 1). Omicron infections were reported to be milder than other strains. Omicron differs from other SARS-CoV-2 strains in three ways: high rate of replication (15), capacity to avoid the humoral immune response, and high rate of reinfection (15). In CML patients, Omicron infection was reported to cause mild infection (16). A similar outcome was also observed in other groups of patients, such as acute leukemia, polycythemia Vera, essential thrombocythemia, and chronic lymphocytic leukemia (17–19).

**Table 1:** Shows a comparison between the different variants of SARS-CoV-2.

Variant	Timeline and region	Global dominance	Characteristics
Delta	India in December 2020	dominant globally until emergence of the Omicron variant.	High risk of transmission and severe disease and hospitalization.
Alpha	UK in late 2020	globally dominant, until the emergence of the Delta variant	might be associated with greater disease severity.
Beta	South Africa in late 2020	no	immune evasion: convalescent plasma did not neutralize viral constructs with Beta spike protein
Gamma	Japan and Brazil in December 2020	no	Possible increased transmissibility and an impact on immunity
Omicron	Botswana and South Africa in November 2021	globally dominant variant	High risk of transmission, immune evasion, but low risk for severe disease

Source: https://www.who.int/en/activities/tracking-SARS-CoV-2-variants/

Unfortunately, few studies regarding SARS-CoV-2 virus infection in CML patients have been done after the introduction of vaccines and the emergence of different strains. Our review included 24 studies that were reported before December 2020. Considering that the first vaccine was available in December 2020, it is less likely that studies published before 2021 included patients who received vaccines. The data showed that there was a significant drop in mortality for patients with CML after the vaccination from 11 to 4%, which is a drop by three times in mortality. The improvement in mortality after vaccination could be attributed to several factors. First, it might be related to the different strains that have different virulence; for example, infections with milder strains like Omicron resulted in a lower mortality rate compared to other strains (Table 1). Additionally, the availability of effective treatment and medications with antiviral activity that were not available initially during the first waves. Similarly, ICU admission rates are probably lower due to the effect of vaccination on preventing fatal illness and severe illness (20). Besides vaccination, adherence to preventive measures, start of new medications, and experience with treating COVID-19 (21).

Generally, Interruption of TKI therapy was found to be associated with poor long-term outcomes. The real question is, is TKI continuation during COVID-19 beneficial or harmful? Few studies reported the status of TKI if it was continued during COVID-19 treatment or was held. For patients who had the TKI continued (*n* = 9), there were no mortality or serious adverse events. Few patients reported that TKIs were held due to prolonged QT, namely with imatinib (*n* = 1) and with nilotinib (*n* = 2). One patient out of the nine patients who continued TKI had the dose of imatinib reduced due to interaction with ritonavir, which resulted in an increased effect of imatinib. It seems that the decision to continue or to hold TKI should be individualized and weigh benefits and risks. For patients with expected serious side effects, it might be reasonable to hold TKI for a few days and then reassess the risks and benefits.

Initial reports showed that CML patients on TKI are noted to have a favorable outcome against severe COVID-19, after noticing that patients on TKI including CML have a favorable outcome. *In vitro* studies showed that gilteritinib, nintedanib, and imatinib pose antiviral activity, and they vary in their potency, likely depending on the host-targeted tyrosine kinase by suppressing viral replication (22). Additionally, It was therapeutically possible to use gilteritinib at a concentration that would result in 90% virus suppression (22). TKI might have a protective effect against SARS-CoV-2 through different mechanisms; firstly, TKI inhibits viral cell entry and fusion with cell membranes and the formation of endosomes (23). Also, Imatinib and other TKI inhibit Abl2 protein expression, which is required for viral growth (24). On the other hand, there is a theoretical risk of increased severe infection in CML patients taking TKI due to targeted inhibition of kinases involved in immune cell function. This might result in suppressed cellular immune response that facilitates viral replication.

Generally, patients with cancer usually have poor outcomes with SARS-CoV-2 infection, particularly lung cancer and hematological cancer (25). Moreover, patients with cancer have an increased risk of thrombosis compared to the non-cancer population. CML is not known to be at high risk for thrombosis compared to other cancers like pancreatic and gastric cancers. Compared to other MPNs like polycythemia vera and essential thrombocythemia, CML has a lower risk of thrombotic events (26). The heparinase, which is associated with angiogenesis and the development of cancer, may provide a mechanistic explanation for the decreased thrombosis in CML. When compared to ET and JAK2-positive MPN, CML, and Jak2-negative, MPN expresses less heparinase in the bone marrow and has fewer thrombosis (27). The reported thrombosis in CML is mainly arterial (myocardial infarction, cerebrovascular accidents, and peripheral artery disease) and mostly linked to the use of different TKI more often reported with the second-generation TKIs nilotinib, dasatinib, and ponatinib (28). TKI use is associated with endothelial dysfunction and vascular toxicity that lead to accelerated atherosclerosis and thrombosis (28). However, previous studies demonstrated no significant increased risk of arterial thrombosis (29). Paradoxically, TKI is associated with an increased risk of bleeding that is related to TKI-associated thrombocytopenia and is seen in dasatinib more than the other TKIs and platelet dysfunction (30). Platelet dysfunction with TKI use was demonstrated with impaired platelet aggregation with epinephrine *in vitro* (31). Overall, TKIs are associated with bleeding and an increased risk of arterial thrombosis; this might explain the lower risk of thrombotic events in CML patients. Considering their increased risk of thrombosis compared to the general population, CML patients with COVID-19 interestingly are not reported to develop additional risk thrombotic events as might be expected. This finding is supported by the Sweden study (32), the study with the longest follow-up for patients with CML and COVID-19, starting from March 2020 to April 2023, showed that there is no significant adverse outcome when compared imatinib to the newer TKI, also the study showed CML patients with no vaccination, had a slightly higher risk of hospitalization compared to their controls (32). The study also raised the finding that few events were reported among these patients. Our review showed that the mortality rate in CML covid patients improved significantly in the post-vaccination era. This could be explained by the improvement of our understanding of COVID-19, the protective effect of vaccines, and the change in the virulence of the recent strains. A similar finding is seen in a systematic review comparing COVID-19 among hematological malignancies; most cases before the vaccination were available (March 10, 2021) showed that CML patients reported no dyspnea, diarrhea, or respiratory distress compared to other cancers like ALL, MPN lymphoma (33).

In this review, there are important limitations; the testing of CML patients for COVID-19 may change from region to region, where patients with mild disease are less likely to be diagnosed with COVID-19 in resource-limited settings, creating a potential bias that results in higher-than-actual mortality rates among these cases as mild cases are not reported. Additionally, the indications for COVID-19 testing changed multiple times and may not have been the same before and after vaccination, which creates a bias when comparing the two eras in terms of mortality and severe disease. Moreover, the availability of vaccines was not synchronous throughout the world, and some of the reported patients in the post-vaccine era may not have had vaccines locally available. On the other hand, during the initial pandemic, there were no clear treatment guidelines. Many studies have tried to find an effective treatment that can reduce the mortality of COVID-19. Currently, there are many medications that have been shown to prove survival benefit in patients with severe COVID-19 pneumonia (32).

## Conclusion

The COVID-19 mortality rates among CML patients did not appear to be extremely high compared to the general population and were lower than most reports calculating COVID-19 mortality among patients with more severe hematologic diseases. Our review showed that the mortality of CML patients with COVID-19 has significantly improved from 11% in the pre-vaccination period to 4% in the post-vaccination era. However, for CML patients with severe infection requiring ICU admission, the mortality remains high. The available data indicates that COVID-19’s effect on patients with chronic myeloid leukemia (CML) still needs to be better understood due to the limited data.

## Data availability statement

The original contributions presented in the study are included in the article/Supplementary material, further inquiries can be directed to the corresponding author.

## Author contributions

EA: Conceptualization, Data curation, Investigation, Methodology, Project administration, Validation, Visualization, Writing – original draft, Writing – review & editing. AA-S: Data curation, Investigation, Writing – review & editing. QA-m: Data curation, Writing – review & editing. ES: Validation, Writing – review & editing, Data curation. MK: Supervision, Validation, Writing – review & editing. KP: Writing – review & editing, Validation, Visualization. AB: Writing – review & editing, Validation. AA: Validation, Writing – review & editing, Visualization. MY: Writing – review & editing, Conceptualization, Data curation, Investigation, Project administration, Supervision.
